# Serum thyroid-stimulating hormone and interleukin-8 levels in boys with autism spectrum disorder

**DOI:** 10.1186/s12974-017-0888-4

**Published:** 2017-06-02

**Authors:** Sarika Singh, Umar Yazdani, Bharathi Gadad, Sayed Zaman, Linda S. Hynan, Nichole Roatch, Claire Schutte, C. Nathan Marti, Laura Hewitson, Dwight C. German

**Affiliations:** 1 Department of Psychiatry, University of Texas Southwestern Medical Center, 5323 Harry Hines Blvd., Dallas, TX 75390-9070 USA; 2 Department of Clinical Sciences, University of Texas Southwestern Medical Center, 5323 Harry Hines Blvd., Dallas, TX 75390 USA; 3 The Johnson Center for Child Health and Development, 1700 Rio Grande St., Austin, TX 78701 USA; 4 Abacist Analytics, LLC, Austin, TX 78701 USA; 5 Present Address: Toxicology Division, CSIR-CDRI, Lucknow, 226021 Uttar Pradesh India

**Keywords:** Autism, Biomarker, Serum proteins

## Abstract

**Background:**

Autism spectrum disorder (ASD) affects approximately 1 in 68 children in the USA. An ASD blood biomarker may enable early diagnosis and/or identification of new therapeutic targets. Serum samples from ASD and typically developing (TD) boys (*n* = 30/group) were screened for differences in 110 proteins using a multiplex immunoassay.

**Results:**

Eleven proteins were found that together could confirm ASD with modest accuracy using multiple training and test sets. Two of the 11 proteins identified here were further tested using a different detection platform and with a larger sample of ASD and TD boys. The two proteins, thyroid-stimulating hormone (TSH) and interleukin-8 (IL-8), have been previously identified as putative biomarkers for ASD. TSH levels were significantly lower in ASD boys, whereas IL-8 levels were significantly elevated. The diagnostic accuracy for ASD based upon TSH or IL-8 levels alone varied from 74 to 76%, but using both proteins together, the diagnostic accuracy increased to 82%. In addition, TSH levels were negatively correlated with the Autism Diagnostic Observation Schedule subdomain scores.

**Conclusions:**

These data suggest that a *panel of proteins* may be useful as a putative blood biomarker for ASD.

## Background

Autism spectrum disorder (ASD) is a neurodevelopmental disorder characterized by deficits in social communication, social interaction, and restricted, repetitive patterns of behavior, interests, or activities [1]. ASD is the fastest growing developmental disability, affecting more children than cancer, diabetes, and AIDS combined. It affects 1 out of every 68 children in the USA, and it is more often found among boys than girls [2]. Many genes have been identified that are related to the disorder and even de novo mutations have been found to occur [3]. However, there is great genetic heterogeneity in ASD as recently shown in 85 quartet families where the majority of the siblings with ASD (70%) did not share the same genetic mutation [4]. While ASD appears to be on the rise, it is unclear whether the growing number of diagnoses reveals a real increase or comes from improved detection and/or changes to diagnostic criteria. Current diagnostic methods and screening tools are somewhat subjective and are difficult to assess in younger children, which can often result in missed opportunities for early intervention. A biological marker that could predict ASD risk, assist in early diagnosis, or even identify potential therapeutic targets would have great clinical utility [5].

While biomarker research in ASD has greatly increased in recent years [6–10], progress has been limited by a number of factors, and no universal biological markers for ASD have yet been identified. One of the biggest issues in developing biological markers for ASD is the heterogeneity of the disorder. There is wide variation in symptoms among children with ASD, and this is further complicated by a number of co-morbid factors associated with the disorder [11]. The present study used serum samples to investigate the levels of a panel of proteins, as a possible diagnostic biomarker for ASD, and the tests were conducted in a gender-specific manner since the disorder is approximately four times more common in males [12]. We have previously reported that a panel of serum proteins can be used to accurately identify patients with Alzheimer’s disease [13], and this approach has been used by others in seeking a blood test for ASD [8, 10, 14, 15]. These latter studies have discovered both inflammatory proteins (e.g., interleukin-8, IL-8) and hormones (e.g., thyroid-stimulating hormone, TSH) differ among children with ASD in both neonatal and pediatric samples. Here, we have used two different research platforms, Myriad Rules-Based Medicine (RBM) and Meso Scale Discovery (MSD), to begin to discover and validate a serum biomarker panel for ASD.

## Methods

### Ethics

The study protocol was submitted by The Johnson Center for Child Health and Development (Austin, TX) and approved by the Austin Multi-Institutional Review Board (AMIRB). All methods employed in the study were carried out in accordance with the relevant guidelines and regulations. Informed consent was obtained from the parents or legal guardians of all subjects prior to their participation in this research.

### Study subjects

The initial study participants used to measure analytes on the RBM platform consisted of 30 boys with ASD and 30 typically-developing (TD) boys, ages 2–8 years. An additional group of 13 ASD and 9 TD samples (boys, ages 2–8 years) were included in the subsequent analysis on the MSD platform to increase the sample size. Subjects were either recruited directly from The Johnson Center clinic, or through the use of informational study flyers circulated around Austin, TX. Written informed consent was received from the parent or guardian of all subjects prior to enrollment. Briefly, the psychiatric, medical, and family histories of all participants were obtained. For the ASD group, the subjects were assessed by a psychologist trained in research reliability using both the Autism Diagnostic Observation Schedule (ADOS) and the Autism Diagnostic Interview–Revised (ADI-R). Clinical diagnosis was made based on these data and overall clinical impression using DSM-IV criteria. For this particular study, subjects with a diagnosis of Asperger’s Syndrome or Pervasive Developmental Disorder––not otherwise specified, were excluded. For the TD group, all subjects underwent a developmental screening using the Adaptive Behavior Assessment System-Second Edition (ABAS‐II) that was assessed by the psychologist. TD subjects were excluded if their score on the ABAS-II suggested possible abnormal development and the need for further evaluation. TD subjects were also excluded if they had a first- or second-degree relative diagnosed with ASD. Subjects diagnosed with a genetic, metabolic, or other concurrent physical, mental, or neurological disorder were excluded, as were subjects that were currently taking psychiatric medications (or had taken psychiatric medications within the last 3 months prior to enrollment). All subjects were healthy with no reported illnesses for 3 weeks prior to participation in the study.

Due to the high degree of phenotypic heterogeneity in ASD, we further sub-characterized ASD subjects into three groups [16]: (i) those who were nonverbal, (ii) those with gastrointestinal (GI) concerns, and (iii) those with regressive autism. Subjects with ASD were defined as nonverbal if there was a complete absence of intelligible words at time of diagnostic assessment of autism. ASD subjects were classified as having GI concerns if they reported at least one of the following symptoms: (i) constipation; (ii) diarrhea; (iii) abdominal bloating, discomfort, or irritability; (iv) gastroesophageal reflux or vomiting; and/or (v) feeding issues or food selectivity. ASD subjects were classified as having no-regression if the child exhibited traits of autism from infancy, and regressive autism if they had typical early development and later lost function in language and/or social interactions (based on questions probed in the ADI-R). The correlations between protein levels, phenotypic sub-groups, and clinically relevant quantitative traits from the ADOS were analyzed.

### Blood collection/storage

A fasting blood draw was performed on healthy children between the hours of 8–10 a.m. Blood was collected in a 3.5 ml serum separation tube (SST; Vacutainer System; Becton-Dickinson) using standard venipuncture technique. The blood was gently mixed in the SST by five inversions and then stored upright for clotting at room temperature for 10–15 min. Blood was then spun immediately after the clotting time in a swing bucket rotor for 15 min at 1100–1300 g at room temperature. Serum was removed immediately after centrifugation and transferred into coded cryovials in 0.5 ml aliquots. Aliquots of serum were immediately placed upright into storage boxes in a −20 °C freezer for up to 6 h. Samples were then transferred to a −80 °C freezer for long-term storage.

### Analyte measurements on RBM platform

Sample aliquots were coded and shipped on dry ice to Myriad Rules-Based Medicine (RBM; Austin TX) for evaluation using DiscoveryMAP 175+ for quantitative immunoassay of inflammatory molecules and hormones. This multianalyte Luminex profiling platform examined over 175 protein analytes [17, 18]. Final data were reported as the absolute concentrations in the serum. A total of 30 ASD and 30 TD male serum samples were analyzed. Some coded duplicate samples were also run and used to analyze analyte measurement performance. Analyte measurements that showed >15% variance were excluded from the data analysis.

### Measurements on the MSD platform

We sought to replicate the serum biomarker proteins identified on the RBM platform by subsequently analyzing proteins on a Meso Scales Discovery (MSD) platform [19] run in-house. Compared with the traditional ELISA approach, the MSD platform shows greater sensitivity and is able to reliably detect different proteins across a broad dynamic range of concentrations [20]. The assay is based upon electro-chemiluminescence technology by using specific capture antibodies coated at corresponding spots on an electric wired microplate. This platform was used to measure TSH in 43 ASD boys and 39 TD boys, and IL-8 in 36 ASD boys and 35 TD boys; the two proteins showing the greatest percent difference in the ASD and TD samples run on the RBM platform. We measured the two proteins in samples that were run in duplicate accordingly to the manufacturer’s protocol. Any duplicate value with >15% variance was removed from the final data analysis, and every plate was run with a standard concentration curve.

### Statistical analyses

The RBM data were analyzed using random forest methods. Random forest analysis was developed as an ensemble learning method that utilizes a classification tree as the base classifier [21]. Hundreds of training and test sets, of 15 subjects/group, were analyzed to determine the importance of a panel of analytes to correctly identify ASD subjects. For the MSD data, differences between the ASD and TD groups were analyzed with Mann-Whitney *U* tests. For comparing the accuracy of the two analytes for predicting ASD vs. TD, we used cut scores and area under the curve (AUC) analyses. Diagnostic accuracy and AUC were computed by ROC (receiver operation characteristic) curves using SPSS V23; the optimum probability cutoffs were determined using mathematical formulas in Microsoft Excel™ to maximize accuracy and the perpendicular distance from the 45 degree line of equality. The *p* < 0.05 level was considered to be statistically significant for analyses using the MSD platform (i.e., for TSH and IL-8 assays).

Regression analyses for ASD subjects were conducted using the R *lavaan* package, which fits models using full information maximum likelihood estimation that makes use of all available data. Thus, data from all ASD subjects were included in each model [22] (*n* = 43 for TSH and *n* = 36 for IL-8). Protein levels were regressed on each of the ADOS subdomain scores and phenotypic sub-grouping to examine whether levels of TSH and/or IL-8 were related to a clinical measure of ASD and comorbidities. Prior to fitting regression models, IL-8 was log-transformed to reduce the positive skew; the transformed distribution was approximately normally distributed and met guidelines for covariance matrix-based models [23].

## Results

### Proteins measured on the RBM platform

A total of 184 analytes were measured in serum samples from 30 ASD boys and 30 TD boys on the RBM Luminex platform; however, 51 analytes were undetectable, and 23 exhibited >15% spot-to-spot variance, and were therefore omitted from analysis. The undetectable proteins likely represent faulty antibodies, as these proteins have been detected with previous versions of RBM's Discovery Map. Eleven of the remaining 110 serum proteins measured, shown in Table 1, were selected based upon a random forest analysis, and 5 of the analytes were significantly different between ASD and TD at the *p* ≤ 0.04 level (uncorrected for multiple comparisons). Using a random selection of 15 ASD and 15 TD subjects for a training set and the same number of subjects for a test set, 100 comparisons of data indicated that the random forest test set could accurately confirm ASD vs. TD samples with an average area under the ROC curves of 0.761. Those proteins with the highest importance for confirming a diagnosis of ASD include TSH, stem cell factor, monocyte chemotactic protein 4, ferritin, and IL-8.

**Table 1:** ASD-related proteins identified using the RBM platform. Data from 30 ASD boys and 30 TD boys

Protein	% Change	*t* test	Importance
Alpha 1 microglobulin (A1Micro)	9%↑	0.017	2.309
Apolipoprotein E (ApoE)	22%↑	0.035	−1.865
Apolipoprotein H (ApoH)	15%↑	0.103	4.153
AXL receptor tyrosine kinase (AXL)	11%↑	0.059	−0.233
Chromogranin A (CgA)	21%↑	0.050	−0.418
Ferritin (FRTN)	29%↑	0.056	3.295
Interleukin 8 (IL-8)	31%↑	0.043	2.568
Monocyte chemotactic protein 4 (MCP4)	18%↑	0.064	3.393
Monokine induced by gamma interferon (MIG)	26%↑	0.166	2.088
Stem cell factor (SCF)	16%↑	0.008	4.356
Thyroid-stimulating hormone (TSH)	31%↓	0.003	14.639

### Proteins measured on the MSD platform

We chose to replicate the findings on the RBM platform on a different platform to be certain of the validity of the RBM findings. We also wished to measure two of the proteins with “high importance” based upon the random forest analysis of the RBM data. We therefore used the MSD electro-chemiluminescence platform to measure levels of TSH and IL-8. All of the samples run on the RBM platform (*n* = 30/group) were also run on the MSD platform, plus some additional samples included to increase the sample size.

**Fig. 1:**
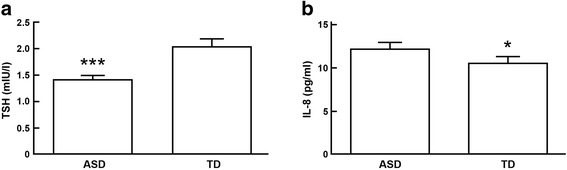
Serum protein measurements on the MSD platform. Data represent mean ± SEM. **a** TSH levels are significantly lower in ASD boys (*p* = 0.007), and **b** IL-8 levels are significantly higher in ASD boys (*p* = 0.025)

Using 43 ASD samples, we found TSH levels to be 30% lower vs. TD samples (*n* = 37): 1.42 ± 0.08 (mean ± SEM) and 2.04 ± 0.16 mIU/l, respectively, (*p* < 0.0056; Mann-Whitney *U* test). TSH levels were within the normal range for this hormone for children of the age studied (http://www.mayomedicallaboratories.com/test-info/pediatric/refvalues/) (see Fig. 1). For the prediction of ASD, TSH levels were 94% sensitive and 60% specific. The area under the ROC curve was 0.674, *p* = 0.006. We measured IL-8 levels in 36 ASD boys, and it was 16% higher vs. TD boys (*n* = 35): 12.17 ± 0.52 (mean ± SEM) and 10.52 ± 0.51 pg/ml, respectively, (*p* < 0.0306; Mann-Whitney *U* test) (see Fig. 1). For the prediction of ASD, IL-8 levels were 94% sensitive and 55% specific. The area under the ROC curve was 0.652, *p* = 0.023.

In order to determine whether the accuracy in confirming ASD vs. TD is enhanced by an analysis using a combination of analytes, we analyzed the prediction accuracy using both TSH and IL-8 analytes (Table 2). Here, we used samples from subjects that were run for both analytes on the MSD platform, which resulted in 18 ASD and 20 TD boys. The predictive accuracy for TSH alone was 76%, and for IL-8 alone, it was 74%. Using the two analytes together, the predictive accuracy was 82%, with 89% sensitivity and 75% specificity. ASD cases were predicted, using cut scores, as having TSH levels below 1.8 mIU/l and IL-8 levels above 10.3 pg/ml. The area under the ROC curve for the model using the two analytes was 0.842 ± 0.067 SEM (*p* < 0.001).

**Table 2:** Descriptive statistics for ASD predictive accuracy of TSH and IL-8

Models	Best cut score	*χ* ^2^	*p*	Accuracy
TSH alone (low is ASD)	1.865	12.48	<0.001	29/38 (76%)
IL-8 alone (high is ASD)	10.345	10.72	0.001	28/38 (74%)
Using above cut scores for TSH and IL-8 for group membership	1.5	15.67	<0.001	31/38 (82%)
LR model both TSH and IL-8	0.61185	14.616	<0.001	30/38 (79%)

Data from 18 ASD and 20 TD boys

*LR* logistic regression

### Association between protein levels and ADOS subdomains

TSH was regressed on each of the ADOS subdomain scores. The following three domains exhibited a significant negative correlation whereby higher scores (more severe symptoms) in the subdomains were associated with lower levels of TSH: social interaction (*z* = −2.61, *p* = 0.009), communication + social interaction (*z* = −2.12, *p* = 0.034), and stereotyped behavior and restrictive interests (SBRI) (*z* = −2.28, *p* = 0.023). There was not a significant relationship between TSH and the ADOS communication subdomain (*z* = −0.55, *p* = 0.581).

IL-8 was regressed on each of the ADOS subdomain scores. Among ADOS subdomains, there were no significant relationships between IL-8 and communication (*z* = 0.16, *p* = 0.871), social interactions (*z* = −0.15, *p* = 0.877), communication + social interactions (*z* = −0.06, *p* = 0.953), or SBRI (*z* = 0.75, *p* = 0.455).

### Association between protein levels and phenotypic data

The percentage of children with ASD who were nonverbal was 50%. The percentage of children with ASD displaying GI issues was 85%. Regressive autism was seen in 63% of the study group. There were no significant relationships between either TSH or IL-8 and the autism sub-groups. For TSH: nonverbal (*z* = −0.51, *p* = 0.609), GI concerns (*z* = −0.14, *p* = 0.890), and regression (*z* = −1.12, *p* = 0.265). For IL-8: nonverbal (*z* = −0.20, *p* = 0.843), GI concerns (*z* = −0.21, *p* = 0.833), and regression (*z* = −0.49, *p* = 0.624).

## Discussion

The goal of the present study was to identify serum proteins that were differentially expressed in ASD and TD samples. Eleven proteins were found that together predicted ASD with modest accuracy. Two of the proteins, TSH, and IL-8 have been identified as ASD biomarkers in previous studies [8, 24, 25], so both the RBM and MSD platforms were used to validate these findings. As previously found, TSH levels were significantly lower in ASD boys whereas IL-8 levels were significantly elevated compared to TD boys. The diagnostic accuracy for predicting ASD based upon the TSH or IL-8 levels alone varied from 74 to 76%, but using both proteins together, the diagnostic accuracy increased to 82%. These data suggest that a panel of proteins may be useful as a blood biomarker for ASD.

Using a quantitative immunoassay for inflammatory molecules and hormones, 11 proteins were found, which when combined, could discriminate serum samples from ASD and TD boys. Among these 11 proteins, TSH, IL-8, alpha 1 microglobulin, apolipoprotein E, and stem cell factor exhibited the highest significant differences between the two groups. TSH levels were significantly lower in the ASD boys compared to TD boys, and based upon the random forest analysis, TSH had the highest *importance* among the panel of 11 analytes for predicting ASD vs. TD. When TSH was studied on the MSD platform, again, the ASD boys had an average 30% lower level vs. TD boys. We limited our study to boys because several studies report sex-specific differences in putative ASD biomarkers [15, 26, 27], and we only had access to a small sample of ASD and TD girls.

TSH is a pituitary hormone that stimulates the thyroid gland to produce thyroxine (T_4_) and then triiodothyronine (T_3_), which stimulates the metabolism of almost every tissue in the body [28]. TSH is secreted throughout life but reaches high levels during the periods of rapid growth and development. The hypothalamus produces thyrotropin-releasing hormone (TRH), which stimulates the pituitary gland to produce TSH. Thyroid hormones are essential for brain maturation and for brain function throughout life. Thyroid hormone deficiency, even for short periods, may lead to irreversible brain damage, the consequences of which depend on the specific timing of onset and duration of thyroid hormone deficiency [28–31].

Reductions in TSH have been reported previously for ASD children. Significantly reduced levels of TSH, and reduced TSH response following TRH stimulation, have been observed by Hashimoto and colleagues [32], where they examined 41 ASD boys (average age of 5.7 years) compared to 5 TD boys (average age of 8.7 years). They also examined 12 boys with mental retardation and 12 boys with minimal brain dysfunction, and their TSH levels were like those of the TD boys. More recently, the RBM platform was used to demonstrate altered levels of 15 blood proteins in ASD, and one of the proteins was TSH [8]. Reduced levels of TSH were observed in blood spots from infants who later were found to have ASD (*n* = 16 ASD and *n* = 32 TD; gender not reported), suggesting that TSH levels may be useful as *an early biomarker for ASD*. These data indicate that the reduced TSH was present at birth. More recently, maternal mid-pregnancy serum TSH levels were found to be inversely correlated with the likelihood of having a child with ASD [33]. This study used 149 control children and 78 ASD children, and both genders were in the two groups.

When ADOS subdomain scores were compared with TSH levels, there was a significant negative correlation with social interaction, communication + social interaction, and SBRI such that a higher subdomain score (i.e., more ASD symptoms) was correlated with lower TSH levels. These data suggest that TSH may not only serve as an important member of an ASD biomarker panel, but it may also represent a useful index of an ASD phenotype.

Proinflammatory cytokines (PICs) have been reported to be elevated in ASD children [34]. In the present study using both the RBM and MSD platforms, we found 17–30% increases in IL-8 in the serum of ASD boys. No changes in levels of IL-10, IL-16, or IL-18 were found in the present study, and no such changes were reported in another study as well [8]. In the study of Mizejewski et al. [8], also run on the RBM platform, elevations in IL-8 were reported in ASD children in blood spots collected at birth, suggesting that IL-8 levels may be useful as an *early biomarker* for ASD. PICs are directly linked with neuroinflammation. Elevations in plasma IL-8 (33%) have also been reported by Suzuki et al. [24] in high-functioning ASD boys, with a mean age of 12 years (*n* = 28 ASD boys and *n* = 28 TD boys). Finally, in a meta-analysis of three studies, Masi et al. [25] report significant elevations in IL-8 in 150 ASD vs. 140 TD children (primarily boys). There was no significant relationship between ADOS and IL-8 scores or between protein levels and ASD sub-groups (nonverbal, GI concerns, and regression). These data suggest that IL-8 levels are not specific to the subdomains used to diagnose ASD.

There are some limitations in this study. The relatively small sample size for our discovery study run on the RBM platform renders the data presented here preliminary, and a larger study with more ASD subjects is planned. However, the sample size is appropriate for the validation of TSH and IL-8 as ASD biomarkers. The increased prevalence of ASD in boys resulted in the study primarily focusing on boys, which does not allow one to thoroughly investigate gender-specific differences. Examination of TSH and IL-8 in ASD and TD girls should be further evaluated in a larger study. When making electro-chemiluminescent measurements on 96-well plates, there are often plate-to-plate differences that add variability to the data; however, we routinely ran standards on every plate as well as calibration curves to minimize this source of data variability.

## Conclusions

In order to identify ASD at an early age and facilitate interventions before symptoms manifest, robust biomarkers are important. The present study validated the finding that levels of TSH are significantly lower in ASD boys vs. TD boys, and we found that the levels were highly correlated with ADOS subdomain scores, suggesting that TSH levels may be useful for assessing specific ASD phenotypes. We also validated IL-8 as an ASD biomarker. It is interesting that when we analyzed the accuracy of predicting ASD vs. TD using more than one analyte, we found that the accuracy went from 74–76% for single analytes to 82% when using TSH and IL-8 together. These data suggest that information on hormone status and inflammation together provide greater diagnostic accuracy for the identification of ASD. The use of *panels of blood proteins* for disease identification and/or characterization appears to be a useful strategy, and one that we will pursue by (i) testing a larger set of ASD and TD samples on the MSD platform, (ii) looking at a total of four analytes previously identified in the RBM platform (e.g., apolipoprotein E and stem cell factor along with TSH and IL-8) to determine whether four protein analytes combined will provide an accuracy of ~90% in predicting ASD in boys or an ASD phenotype in a subgroup, and (iii) investigating the levels of these analytes in blood samples from much younger children that then went on to develop ASD.

## References

[1] American Psychiatric Association. Diagnostic and Statistical Manual of Mental Disorders (5th ed.) Washington, DC; Author; 2013.

[2] CDC (2014). Prevalence of autism spectrum disorder among children aged 8 years—autism and developmental disabilities monitoring network, 11 sites, United States. MMWR.

[3] O'Roak BJ, Stessman HA, Boyle EA, Witherspoon KT, Martin B, Lee C, Vives L, Baker C, Hiatt JB, Nickerson DA (2014). Recurrent de novo mutations implicate novel genes underlying simplex autism risk. Nat Commun.

[4] Yuen RK, Thiruvahindrapuram B, Merico D, Walker S, Tammimies K, Hoang N, Chrysler C, Nalpathamkalam T, Pellecchia G, Liu Y (2015). Whole-genome sequencing of quartet families with autism spectrum disorder. Nat Med.

[5] Hewitson L (2013). Scientific challenges in developing biological markers for autism. OA Autism.

[6] Glatt SJ, Tsuang MT, Winn M, Chandler SD, Collins M, Lopez L, Weinfeld M, Carter C, Schork N, Pierce K (2012). Blood-based gene expression signatures of infants and toddlers with autism. J Am Acad Child Adolesc Psychiatry.

[7] Momeni N, Bergquist J, Brudin L, Behnia F, Sivberg B, Joghataei MT, Persson BL (2012). A novel blood-based biomarker for detection of autism spectrum disorders. Transl Psychiatry.

[8] Mizejewski GJ, Lindau-Shepard B, Pass KA (2013). Newborn screening for autism: in search of candidate biomarkers. Biomark Med.

[9] Ngounou Wetie AG, Wormwood KL, Russell S, Ryan JP, Darie CC, Woods AG (2015). A pilot proteomic analysis of salivary biomarkers in autism spectrum disorder. Autism Res.

[10] West PR, Amaral DG, Bais P, Smith AM, Egnash LA, Ross ME, Palmer JA, Fontaine BR, Conard KR, Corbett BA (2014). Metabolomics as a tool for discovery of biomarkers of autism spectrum disorder in the blood plasma of children. PLoS ONE.

[11] Amaral DG (2011). The promise and the pitfalls of autism research: an introductory note for new autism researchers. Brain Res.

[12] Schaafsma SM, Pfaff DW (2014). Etiologies underlying sex differences in autism spectrum disorders. Front Neuroendocrinol.

[13] O'Bryant SE, Xiao G, Zhang F, Edwards M, German DC, Yin X, Como T, Reisch J, Huebinger RM, Graff-Radford N (2014). Validation of a serum screen for Alzheimer’s disease across assay platforms, species, and tissues. J Alzheimers Dis.

[14] Pramparo T, Pierce K, Lombardo MV, Carter Barnes C, Marinero S, Ahrens-Barbeau C, Murray SS, Lopez L, Xu R, Courchesne E (2015). Prediction of autism by translation and immune/inflammation coexpressed genes in toddlers from pediatric community practices. JAMA Psychiat.

[15] Schwarz E, Guest PC, Rahmoune H, Wang L, Levin Y, Ingudomnukul E, Ruta L, Kent L, Spain M, Baron-Cohen S (2011). Sex-specific serum biomarker patterns in adults with Asperger’s syndrome. Mol Psychiatry.

[16] Napolioni V, Ober-Reynolds B, Szelinger S, Corneveaux JJ, Pawlowski T, Ober-Reynolds S, Kirwan J, Persico AM, Melmed RD, Craig DW (2013). Plasma cytokine profiling in sibling pairs discordant for autism spectrum disorder. J Neuroinflam.

[17] Chowdhury F, Williams A, Johnson P (2009). Validation and comparison of two multiplex technologies, Luminex and Mesoscale Discovery, for human cytokine profiling. J Immunol Methods.

[18] Krishhan VV, Khan IH, Luciw PA (2009). Multiplexed microbead immunoassays by flow cytometry for molecular profiling: basic concepts and proteomics applications. Crit Rev Biotechnol.

[19] Belzeaux R, Lefebvre MN, Lazzari A, Le Carpentier T, Consoloni JL, Zendjidjian X, Abbar M, Courtet P, Naudin J, Boucraut J (2017). Measuring blood cytokines in biological psychiatry using commercially available multiplex immunoassays. Psychoneuroendocrinology.

[20] Burguillos MA (2013). Use of meso-scale discovery to examine cytokine content in microglia cell supernatant. Methods Mol Biol.

[21] Breiman L (2001). Random forests. Mach Learn.

[22] Graham J (2012). Missing data: analysis and design.

[23] Curran PJ, West SG, Finch J (1996). The robustness of test statistics to non-normality and specification error in confirmatory factor analysis. Psychol Methods.

[24] Suzuki K, Matsuzaki H, Iwata K, Kameno Y, Shimmura C, Kawai S, Yoshihara Y, Wakuda T, Takebayashi K, Takagai S (2011). Plasma cytokine profiles in subjects with high-functioning autism spectrum disorders. PLoS ONE.

[25] Masi A, Quintana DS, Glozier N, Lloyd AR, Hickie IB, Guastella AJ (2015). Cytokine aberrations in autism spectrum disorder: a systematic review and meta-analysis. Mol Psychiatry.

[26] Spratt EG, Granholm AC, Carpenter LA, Boger HA, Papa CE, Logan S, Chaudhary H, Boatwright SW, Brady KT (2015). Pilot study and review: physiological differences in BDNF, a potential biomarker in males and females with autistic disorder. Int Neuropsychiatr Dis J.

[27] Steeb H, Ramsey JM, Guest PC, Stocki P, Cooper JD, Rahmoune H, Ingudomnukul E, Auyeung B, Ruta L, Baron-Cohen S (2014). Serum proteomic analysis identifies sex-specific differences in lipid metabolism and inflammation profiles in adults diagnosed with Asperger syndrome. Mol Autism.

[28] Morreale de Escobar G, Obregon MJ (2004). Escobar del Rey F. Role of thyroid hormone during early brain development. Eur J Endocrinol.

[29] Anderson GW, Schoonover CM, Jones SA (2003). Control of thyroid hormone action in the developing rat brain. Thyroid.

[30] Bernal J (2005). Thyroid hormones and brain development. Vitam Horm.

[31] Koibuchi N, Iwasaki T (2006). Regulation of brain development by thyroid hormone and its modulation by environmental chemicals. Endocr J.

[32] Hashimoto T, Aihara R, Tayama M, Miyazaki M, Shirakawa Y, Kuroda Y (1991). Reduced thyroid-stimulating hormone response to thyrotropin-releasing hormone in autistic boys. Dev Med Child Neurol.

[33] Yau VM, Lutsky M, Yoshida CK, Lasley B, Kharrazi M, Windham G, Gee N, Croen LA (2015). Prenatal and neonatal thyroid stimulating hormone levels and autism spectrum disorders. J Autism Dev Disord.

[34] Ashwood P, Krakowiak P, Hertz-Picciotto I, Hansen R, Pessah I, Van de Water J (2011). Elevated plasma cytokines in autism spectrum disorders provide evidence of immune dysfunction and are associated with impaired behavioral outcome. Brain Behav Immun.

